# Albumin Microspheres as “Trans-Ferry-Beads” for Easy Cell Passaging in Cell Culture Technology

**DOI:** 10.3390/gels7040176

**Published:** 2021-10-21

**Authors:** Patrizia Favella, Susanne Sihler, Heinz Raber, Ann-Kathrin Kissmann, Markus Krämer, Valerie Amann, Dennis Kubiczek, Jennifer Baatz, Fabian Lang, Fabian Port, Kay-Eberhard Gottschalk, Daniel Mayer, Barbara Spellerberg, Steffen Stenger, Ingrid Müller, Tanja Weil, Ulrich Ziener, Frank Rosenau

**Affiliations:** 1 Institute of Pharmaceutical Biotechnology, Ulm University, Albert-Einstein-Allee 11, 89081 Ulm, Germany; Patrizia.Favella@uni-ulm.de (P.F.); Heinz.Raber@uni-ulm.de (H.R.); Ann-kathrin.kissmann@uni-ulm.de (A.-K.K.); Markus-1.Kraemer@uni-ulm.de (M.K.); Valerie.amann@uni-ulm.de (V.A.); dennis.kubiczek@gmx.de (D.K.); Jennifer.Baatz@alumni.uni-ulm.de (J.B.); fabian.lars.lang@icloud.com (F.L.); 2 Department of Life Sciences, Albstadt-Sigmaringen University of Applied Sciences, 72488 Sigmaringen, Germany; mueller@hs-albsig.de; 3 Institute of Organic Chemistry III—Macromolecular Chemistry and Organic Materials, Ulm University, 89081 Ulm, Germany; Susanne.Sihler@uni-ulm.de (S.S.); Ulrich.Ziener@uni-ulm.de (U.Z.); 4 Institute of Experimental Physics, Ulm University, 89081 Ulm, Germany; fabian.port@uni-ulm.de (F.P.); kay.gottschalk@uni-ulm.de (K.-E.G.); 5 Institute of Medical Microbiology and Hospital Hygiene, Ulm University, 89081 Ulm, Germany; Daniel.Mayer@uniklinik-Ulm.de (D.M.); Barbara.Spellerberg@uniklinik-Ulm.de (B.S.); Steffen.Stenger@uniklinik-ulm.de (S.S.); 6 Max-Planck-Institut für Polymerforschung Mainz, 55128 Mainz, Germany; weil@mpip-mainz.mpg.de

**Keywords:** hydrogel, beads, cell culture, cell transfer

## Abstract

Protein hydrogels represent ideal materials for advanced cell culture applications, including 3D-cultivation of even fastidious cells. Key properties of fully functional and, at the same time, economically successful cell culture materials are excellent biocompatibility and advanced fabrication processes allowing their easy production even on a large scale based on affordable compounds. Chemical crosslinking of bovine serum albumin (BSA) with N-(3-dimethylaminopropyl)-N’-ethylcarbodiimide hydrochloride (EDC) in a water-in-oil emulsion with isoparaffinic oil as the continuous phase and sorbitan monooleate as surfactant generates micro-meter-scale spherical particles. They allow a significant simplification of an indispensable and laborious step in traditional cell culture workflows. This cell passaging (or splitting) to fresh culture vessels/flasks conventionally requires harsh trypsinization, which can be omitted by using the “trans-ferry-beads” presented here. When added to different pre-cultivated adherent cell lines, the beads are efficiently boarded by cells as passengers and can be easily transferred afterward for the embarkment of novel flasks. After this procedure, cells are perfectly viable and show normal growth behavior. Thus, the trans-ferry-beads not only may become extremely affordable as a final product but also may generally replace trypsinization in conventional cell culture, thereby opening new routes for the establishment of optimized and resource-efficient workflows in biological and medical cell culture laboratories.

## 1. Introduction

Hydrogels have become an important class of material in diverse fields of applications such as bulk absorber materials [1,2], cosmetics [3], and even building industries [4]. Especially hydrogels making use of the advantageous intrinsic properties of biopolymers such as proteins and nucleic acids with their defined sequence lengths (also referred to as ‘precision polymers’) have been shown to possess advanced properties over synthetic polymers for life science-related applications, including biochemistry, cell biology and the field of medical applications [5,6,7,8,9]. In the past years, protein hydrogels were developed based on blood-derived proteins as building blocks, with serum albumins either from human or bovine origin being prominent examples. These proteins have crucial advantages for (biotechnological) applications, especially in the context of the field of cell culture/manipulation as they are considered per se biocompatible, in principle non-immunogenic, and have proved their (enzymatic) biodegradability [10,11,12]. An additional advantage is that these proteins are economically extremely affordable, with bovine serum albumin (BSA) being even far less expensive than its human counterpart (HSA). Hydrogels have been developed based on these proteins, involving very simple crosslinking reactions with also very inexpensive chemical crosslinkers such as four-armed amino reactive tetrakis(hydroxymethyl)phosphonium chloride (THPC) to form biocompatible and biodegradable hydrogel matrices [10,11,12,13,14,15,16].

BSA-based hydrogels have already been introduced as so-called affinity layers in anti-infective wound dressings. Here, specific binding properties towards different pathogenic microorganisms were introduced by functionalizing their surfaces with lectin as a sugar-binding affinity entity. Apart from these rather simple examples as potential wound dressings for surgery, their application in more sophisticated technologies has been suggested as well. 3D cell cultures of different cell lines have been accomplished in BSA-based hydrogels by modification with the sugar-binding protein lectin. Lectin allows reversible capturing of cells that then grow in the hydrogel matrix, and cell harvesting proceeds by simple elution with the respective lectin-binding sugars that compete for the lectin-binding site [15,16]. Although elaborate 3D cell culture technologies are currently being developed, their application is still focused on solving specific scientific problems, and they are not applied for routine usage. Instead, cultivation of eukaryotic cells is routinely performed in 2D culture systems, mainly involving polystyrene flasks, dishes, or microtiter plates with or without appropriate coatings allowing growth, especially of adherent cells. Cultivating such adherent cells requires cell growth on surfaces until the cells reach the state of ‘confluency,’ which describes the formation of an almost closed monolayer of cells. Further propagation for subsequent analyses or cell biological experiments requires avoiding cellular overgrowth to exclude potential alterations in cellular physiology or biochemistry of the cell surface due to the loss of productive contacts to the plastic surface as a substratum with defined mechanical and chemical properties or even inhibition of the cells during confluency. Keeping cells growing (ideally in the exponential phase) requires their transfer to fresh surfaces and providing fresh nutrition (i.e., fresh medium and supplements). This cell transfer is usually done by the so-called “splitting” or “passaging” of cells to fresh cultivation vessels at an appropriate time point before cells reach confluency, which needs to be optimized for each cell line. Passaging is only possible when cells are detached from the substratum by the effective loosening of molecular interactions of the cell surface to the growth support, which can be catalyzed by different proteolytic enzymes with trypsin probably representing the ‘gold standard’ of proteases in laboratories worldwide [17]. Moreover, efficient detachment may require additional mechanical forces by scraping the cells from the plastic surface using a ‘rubber policeman’. Both hydrolytic removals of extracellular domains of cellular membrane proteins and robust mechanic treatment pose a significant and severe physiological stress to the cells [17], which can lead potentially to even long-lasting adverse effects on physiological processes and/or regulatory circuits, and the entire process is extremely laborious [18,19]. In addition to these disadvantages, traditional passaging is time-consuming enough that probably a majority of Ph.D. students and technical personnel responsible for maintaining a cell culture laboratory may regard it as an excessively labor intensive job.

**Figure 1:**
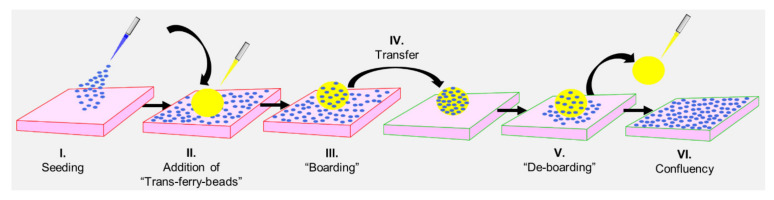
Workflow of the novel trans-ferry-bead mediated transfer of cells (“splitting”/“passaging”) in cell culture. The process can be summarized in six distinct steps involving (**I**.) seeding of cells, (**II**.) addition of trans-ferry-beads to any pre-grown cell culture vessel (microtiter plate, flask, reactor, etc.), (**III**.) providing sufficient time to on-grow and cover the beads (i.e., “boarding”), (**IV**.) transfer of the passenger loaded beads to the destination cell culture vessel followed by (**V**.) settling to the new habitat (i.e., “de-boarding”) and finally (**VI**.) reaching the typical monolayer growth stage on the surface of the new cell culture vessel represented by the so-called confluency.

**Figure A1:**
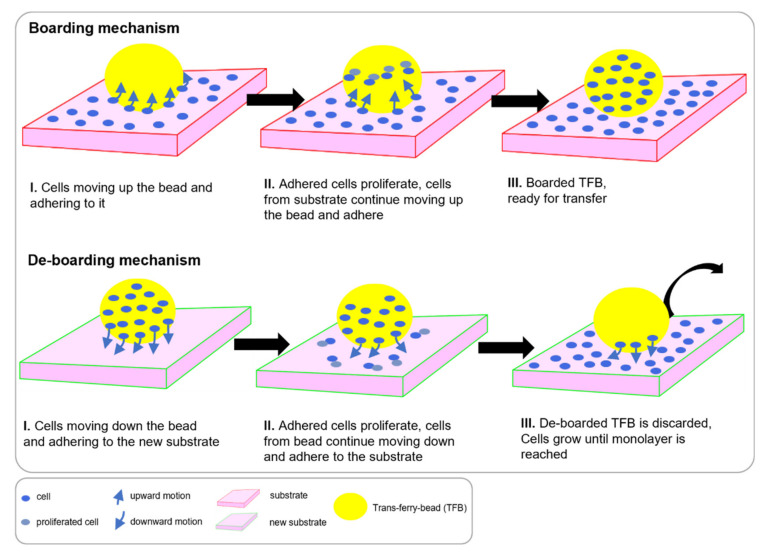
Detailed boarding and de-boarding mechanism of the Trans-ferry-beads. Boarding: The cells board the empty bead and proliferate. Cells from substrate surface continue the boarding process until the bead is ready for transfer. De-boarding: The cell-loaded bead is transferred to a new substrate. Cells de-board, adhere to a new substrate surface and proliferate. The de-boarding process is completed when all cell passengers have left the Trans-ferry-bead.

**Figure A2:**
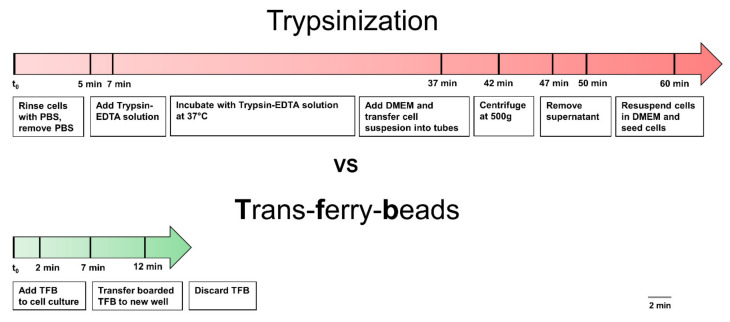
Trypsinization vs. Trans-ferry-beads. Comparison of working steps of the traditional splitting method and new method with Trans-ferry-beads.

Herein, we have designed a robust and stable BSA-based hydrogel matrix providing cell adhesive properties for fast and easy passaging of different cell lines. The biogel could be sterilized and kept under sterile conditions required for in vivo applications, and it is produced in a cost-effective fashion. BSA molecules were covalently crosslinked after activating the carboxylic acid surface groups and intermolecular bioconjugation with the primary amino groups of neighboring BSA molecules. Performing this crosslinking reaction in an inverse (water-in-oil) emulsion with the petroleum distillate IsoparM™ (isoparaffinic oil) mixed with sorbitan monooleate (Span™ 80) delivered BSA hydrogel beads with almost perfect spherical shapes and diameters tunable from 400–950 µm simply by varying the stirring speed. The reaction in the emulsion can be expected to allow upscaling of the process into even industrially relevant production scales, which is a promising pre-condition for the development of an economically feasible final product. When added to cell cultures of a lung cancer cell line (A549) and different fibroblast cell lines from rat, monkey, and human, the resulting chemically sterilized beads could serve as a “mobile” substratum for these cells [20]. The beads allowed the transfer of a cell population from one cell culture vessel to the next, leading to an efficient embarkment of this new habitat (Figure A1). The term “trans-ferry-beads” refers to the property of the protein hydrogel beads transporting cells between old and new culture vessels with a ferry transferring passengers (Figure 1). This system is cheap and simple to use and offers the potential to replace trypsinization as a harsh methodology for the splitting/passaging in conventional cell culture procedures to provide new avenues for establishing optimized and resource-efficient workflows in biological and medical cell culture laboratories (Figure A2).

## 2. Results and Discussion

Hydrogels fabricated from biological sources such as proteins often termed “biogels”, have emerged as a versatile class of biocompatible and biodegradable materials for various applications such as cell culture technologies [10,11], smart wound dressings [13], and drug delivery [21]. Protein hydrogels based on blood proteins such as bovine or human serum albumin (BSA or HSA) even allow 3D cell culture applications, and they are comprehensively compatible with most cell lines.

### 2.1. Fabrication of Trans-Ferry-Beads

**Figure 2:**
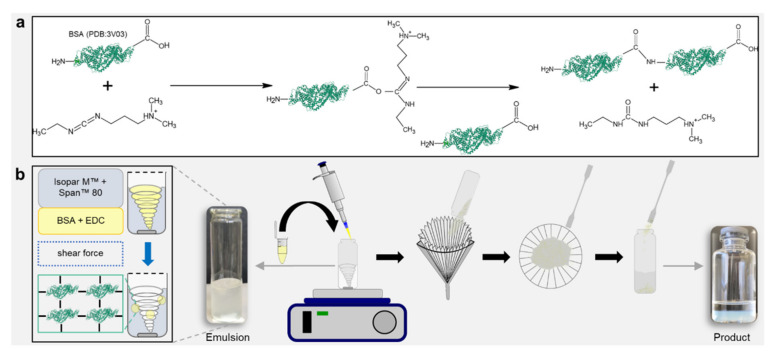
Simplicity of the emulsion-based fabrication process delivering micrometer-scale hydrogel spheres (“trans-ferry-beads”). (**a**) Activation of BSA (PDB:3V03). Crosslinking by activating carboxyl groups of the BSA molecule with EDC, forming a BSA conjugate, and releasing a water-soluble urea derivative by-product. (**b**) Fabrication of hydrogel beads. Emulsion-based reaction process on a magnetic stirrer. Harvesting the beads by filtering, washing, and storing in 70 vol% ethanol.

The biogels in the present contribution were based on BSA crosslinked with the help of N-(3-dimethylaminopropyl)-N’-ethylcarbodiimide hydrochloride (EDC) (Figure 2a). To fabricate hydrogel particles with spherical shapes, the crosslinking reactions were performed in emulsions. The continuous phase consisted of an oil–surfactant mixture, the aqueous phase of the hydrogel mixture. For the generation of the continuous phase of the emulsion, the petroleum distillate Isopar M^™^ (isoparaffinic oil) was mixed with sorbitan monooleate (Span^™^ 80), a surfactant used for water-in-oil emulsions. The aqueous phase consisted of a mixture of BSA and the activating agent EDC in a 2-(*N*-morpholino)ethanesulfonic acid monohydrate (MES) buffer. The oil phase was stirred at 1000, 1250, and 1500 rpm, respectively; the BSA and EDC aqueous solutions were mixed and added to the oil phase immediately in order to guarantee that crosslinking did not start until emulsion droplets formed. Stirring was continued until the gel formation was completed. Crosslinking occurred by activating carboxyl groups of the BSA molecule with EDC, forming an O-acylisourea active ester intermediate, which subsequently reacted with the amino groups of another or the same BSA molecule. The intermediate is then displaced by a nucleophilic attack of the primary amine from another BSA and formed a new amide bond. The water-soluble and non-toxic 1-[3-(dimethylamino)propyl]-3-ethylurea hydrochloride (urea derivative) was released as a byproduct.

After the crosslinking was completed, the beads were harvested from the continuous phase simply by filtering and washing off the residual oil in ethanol (70% (*v*/*v*)). This very simple process offerred opportunities, which may be highly advantageous for the economic success of an aspired potential industrial product for cell culture technology based on our trans-ferry-beads. By simple stirring, the process can be easily scaled up for the use of (bio)reactors for large-scale production. The downstream processing only involves washing with ethanol, which is probably the most common and efficient disinfectant in micro and cell biology and, thus, the process delivers the final product for storage and sterile utilization in the biological or medical cell culture laboratory (Figure 2b).

### 2.2. Characterization of the Trans-Ferry-Beads

**Figure 3:**
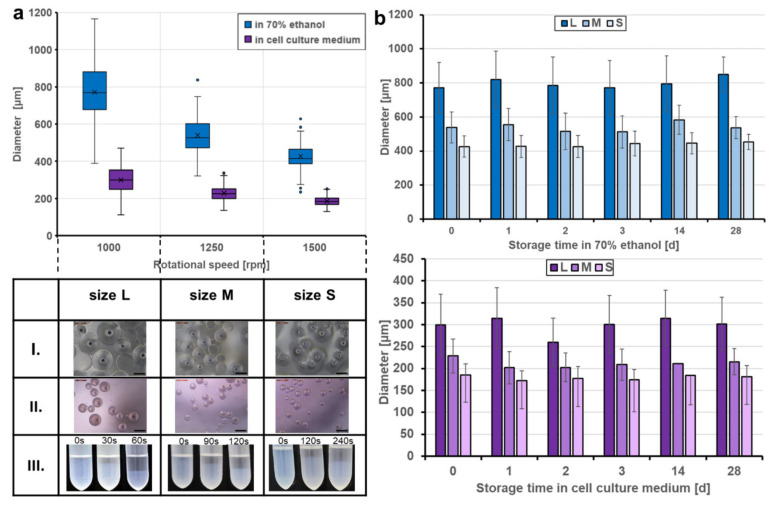
Produced sizes and storage stability of the trans-ferry-beads. (**a**) Fabrication of the beads at rotational speeds of 1000, 1250, and 1500 rpm, creating three different sizes: L, M, and S. Transferring the beads to cell culture medium (DMEM) for equilibration and preparation for cell culture resulted in reproducible size loss. The table shows optical microscopy images (Leica Microsystems GmbH, Wetzlar, Germany) of the three bead sizes stored in 70 vol% ethanol (I. top row), cell culture medium (II. middle row), and the time-dependent sedimentation in ethanol (III. lower row). (**b**) Storage stability in different media. Trans-ferry-beads were stored in 70 vol% ethanol and DMEM for 28 days. The bead size, appearance, and shape were analyzed during the storage period. A factorial ANOVA was used to examine the impact of the storage time on the size (=stability) of the beads. There was a significant impact with a trivial effect size detected in ethanol (F (5) = 13.809, *p* < 0.001, η^2^ = 0.007) and DMEM (F (5) = 25.101, *p* < 0.001, η^2^ = 0.015) showing that there is no relevant loss in stability over the storage time. In ethanol, *n* = 3240 (540 beads per day) and in DMEM, *n* = 3240 (540 beads per day). The average size of the beads, expressed as the mean ± SD, was analyzed with the imaging software FIJI.

**Figure A3:**
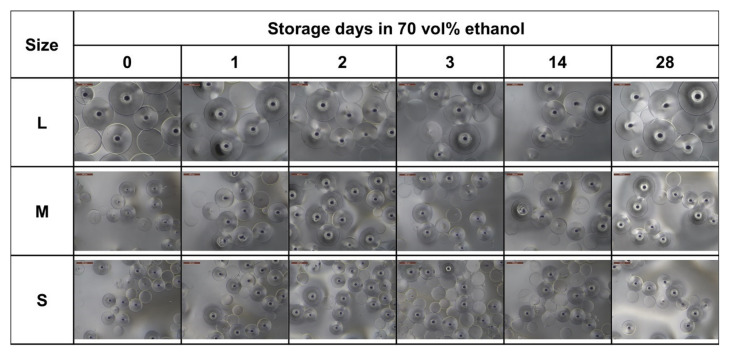
Storage stability in ethanol. Optical microscope images of the three bead sizes stored in 70 vol% ethanol at 4 °C for 28 days.

**Figure A4:**
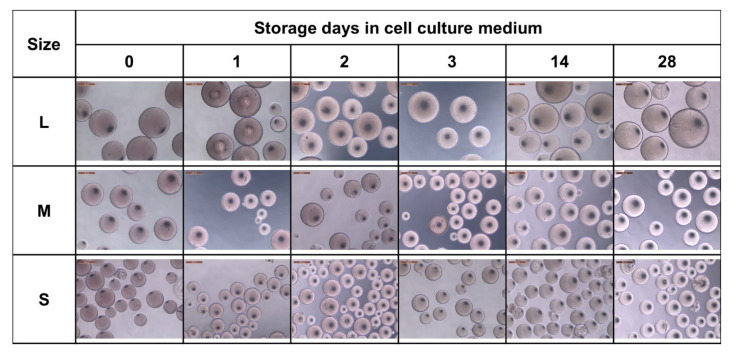
Storage stability in cell culture medium. Optical microscope images of the three bead sizes stored in cell culture medium (DMEM) at 37 °C for 28 days.

The sizes of the resulting beads can be adjusted by the rotational speed during stirring. Applying speeds of 1000, 1250, and 1500 rpm defined three classes of particle size distributions (L, M, S sizes). After harvesting, washing, and storage in ethanol, particle sizes of 772 ± 149 µm (L), 539 ± 91 µm (M), and 463 ± 63 µm (S), respectively, were obtained. Transferring the beads to Dulbecco’s modified eagle medium (DMEM) for equilibration and preparation for cell culture resulted in a considerable and reproducible decrease in particle size (Figure 3a). The medium DMEM contains a cocktail of non-essential amino acids (0.01 M), fetal bovine serum, antibiotics, and significant glucose concentrations (4.5 g L^−1^). The transfer to the higher osmolarity of this medium may result in dehydration of the microspheres and a shrinking of the network due to glucose binding to the crosslinked BSA units, similar to the effect of glucose-dependent contraction of dedicated glucose-responsive smart hydrogels [21]. The degree of shrinking was in the range of approximately 60% for the three classes with 299 ± 70 µm, 228 ± 39 µm, and 185 ± 24 µm of the L, M. and S sizes, respectively. However, the size was sufficiently high to ensure efficient and fast sedimentation in sterile storage solution within seconds (Figure 3a, lower panel III.) as well as in DMEM cell culture medium (not shown), which is an important prerequisite for easy and safe handling of the beads in cell culture experiments. Another important aspect was to equip the trans-ferry-beads with sufficient storage stability with respect to the possibility of long-term storage. Although the experiments to determine the stability over extended periods of several months are still proceeding, the first set of analyses revealed stability of at least four weeks without any sign of degradation or deterioration (Figure A3), which perfectly meets the demands and thus, reasonably promises satisfying storage properties of a final product (Figure 3b). In addition, more importantly, the same was true for trans-ferry-beads in DMEM cell culture medium in which they could perfectly be stored for the same period of time without a considerable change in particle size (i.e., stability) (Figure A4).

### 2.3. Trans-Ferry-Beads Have a Spherical Shape and Are Biocompatible

**Figure 4:**
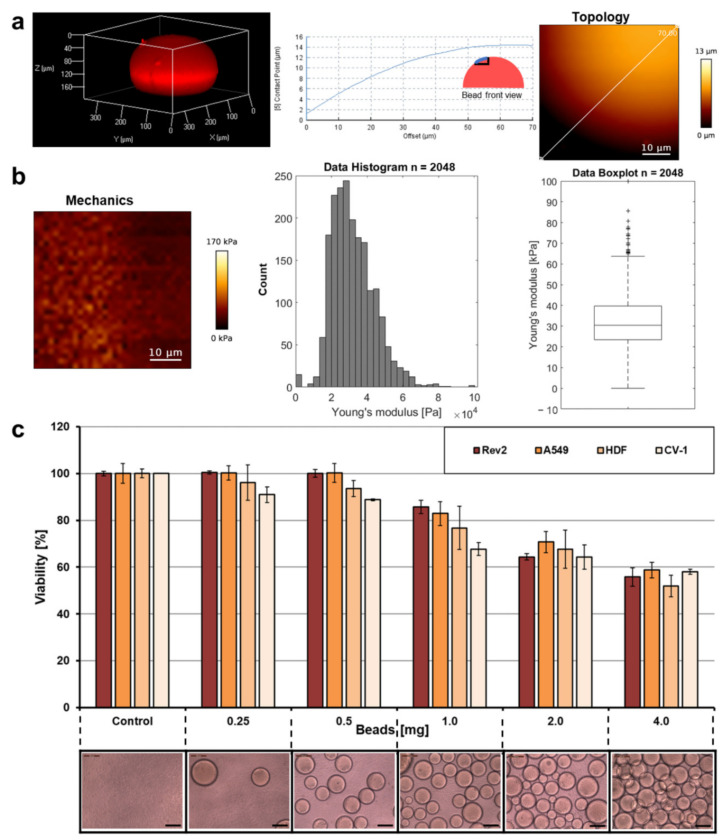
Topology, mechanical properties, and cell compatibility. (**a**) A bead was stained with the fluorescent dye rhodamine B by diffusion and analyzed with a Confocal Laser Scanning Microscope at 561 nm to prove the overall spherical shape of the trans-ferry-beads. Elasticity of the hydrogel beads in DMEM cell culture medium was measured by atomic force microscopy (AFM). AFM height image is shown of a detail of the bead’s surface with cross-section along the straight line. (**b**) AFM Young’s modulus image with histogram and box plot (median 30.42 ± 31.95 kPa). (**c**) A resazurin assay to test the metabolic activity (=viability) of four different adhesive cell lines after 72 h incubation with different quantities of trans-ferry-beads (lower row). All measurements were performed in triplicate. The error bars represent the standard deviation.

The topology and the mechanical properties of representative bead examples were analyzed by atomic force microscopy (AFM) and supported the impression that the beads approximate an overall spherical shape (Figure 4a) and follow an astonishing narrow distribution of Young’s moduli for a porous material such as the protein hydrogel presented here (Figure 4b). The overall stiffness of the spheres is similar to other hydrogel materials we have previously developed for cell culture [10,11,12,16,22] or wound dressing [13,15] applications.

The most important prerequisite for the feasibility of the trans-ferry-bead concept was that the cell (bio)compatibility was as high as possible when cells in cell culture were confronted with the presence of the beads. The principle cell culture compatibility of BSA-derived hydrogels material was proved in previous studies to be non-toxic [11,12,13,15,16,22]. We used the alternative crosslinker EDC and, thus, the toxicity of the trans-ferry-beads can be excluded since a urea derivative as the only by-product is not only non-toxic, but urea is an approved component, for example, in cosmetic applications [3]. Nevertheless, the metabolic activity of cultures of the different adhesive cell lines as measured in a resazurin-based viability assay was inhibited with growing amounts of the beads in the cell culture vessels. This effect was observed when the amount of beads exceeded 0.5 mg per well of the microtiter well and reached an inhibition of approximately 50% when 4 mg were added (Figure 4c). Taking into account the geometry and the average size of the beads and the dimensions of the wells, 4 mg represents an almost complete double layer of beads covering its bottom, whereas a 0.5 mg portion, which proved to be not inhibitory in this set of experiments, consequently represented a complete quarter coverage of the cell culture area of the well bottom. Thus, we assume that the undisputable loss of metabolic activity/viability is a consequence of negative spatial or mechanical influences of the beads on the growing cells rather than toxic effects of the hydrogel material directly intoxicating the cells or of compounds diffusing out of the beads. Based on this, we decided to use the permissive amount of trans-ferry-beads for the following experiments of the study.

### 2.4. Cell Lines Successfully Board and De-Board Trans-Ferry-Beads

**Figure 5:**
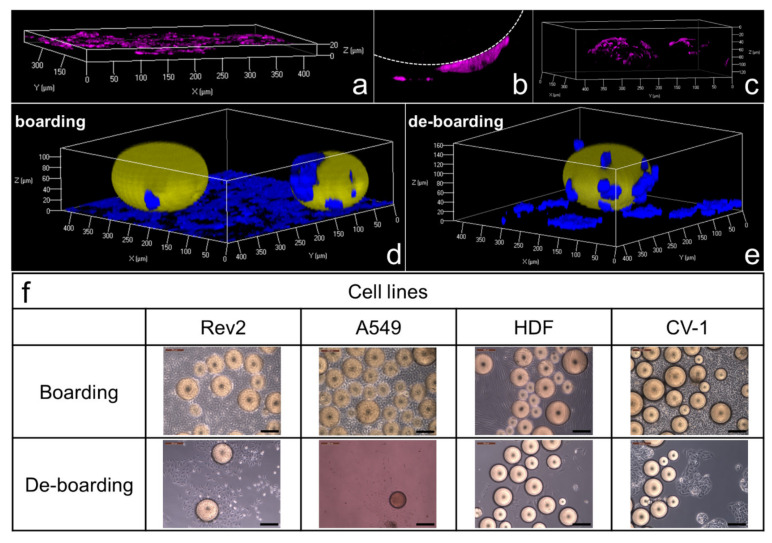
Boarding and de-boarding process of the cell passengers on the trans-ferry-beads. (**a**) Rev2 cells formed a typical 2D monolayer on the plastic material of the microscopic cell culture slide in the absence of the trans-ferry-beads. (**b**) The ‘boarding’ process from the plastic substrate to a trans-ferry-bead (indicated with a dashed line). (**c**) Cells covering the beads reflecting the curvature of the spherical shape of the beads. Cells stained with cell-specific rhodamine-phalloidin. The cells and adhesion areas were investigated with a Confocal Laser Scanning Microscope (CLSM) at 540 nm. (**d**) Boarding and (**e**) ‘de-boarding’ process of Rev2 cells visualized by staining the cells with cell-specific phalloidin Atto 633 and counter-staining the beads with DY495-NHS-Ester. The cells, trans-ferry-beads, and adhesion areas were investigated with a CLSM at 488 and 633 nm, respectively. (**f**) Optical microscopy images illustrating the overall boarding and de-boarding processes for the set of cell lines.

The possibility to grow cells on the beads and to use the hydrogel material as a growth substrate was tested with the Rev2 cell line [23]. It was chosen as a revertant of a rat cancer cell line, and it resembles physiological and behavioral properties of wild-type non-cancer cells, in combination with fluorescent staining of the cytoskeleton using the well-established phalloidin rhodamine conjugate and confocal laser scanning microscopy (CLSM). Whereas Rev2 cells formed a typical 2D monolayer on the plastic material of microscopic cell culture slides in the absence of the trans-ferry-beads (Figure 5a), they appeared to perceive a considerable amount of attraction by the beads, which was high enough to make them grow from plastic well-plate to the hydrogel material. This ‘boarding’ from plastic to a trans-ferry-bead is exemplified in Figure 5b and typically leads to coverage of the beads within 48 h, perfectly reflecting the curvature of the spherical shape of the beads in the microscopic analyses (Figure 5c). Subsequently, when the boarding was complete, both trans-ferry-beads and the cell passengers were transferred to fresh conventional cell culture vessels, and it was analyzed whether the desired tendency of cells to leave the trans-ferry-beads and to adhere again to the plastic material and start growing really existed. Thus, the boarding and ‘de-boarding’ were visualized by CLSM in combination with phalloidin Atto 633 staining of the cells and counter-staining of the beads with a DY495-NHS-ester (Figure 5d,e) and transmitted light microscopy (Figure 5f). While Figure 5d,e may serve as snapshot-like documentations of cells still adhering to the plastic material and at the same time already starting to occupy the bead surface (Figure 5d) or vice versa (Figure 5e), the panel of images in Figure 5f illustrates the overall success of the boarding and de-boarding processes. Moreover, in addition to the results with the Rev2 cell line, the functionality of the trans-ferry-bead concept became obvious also for A549 [20], Human-Dermal-Fibroblasts [24], and CV-1 [25] cells as further members of our set of different cell lines.

### 2.5. Bead-Mediated Passaging Does Not Reduce Cell Fitness and Growth Behavior

**Figure 6:**
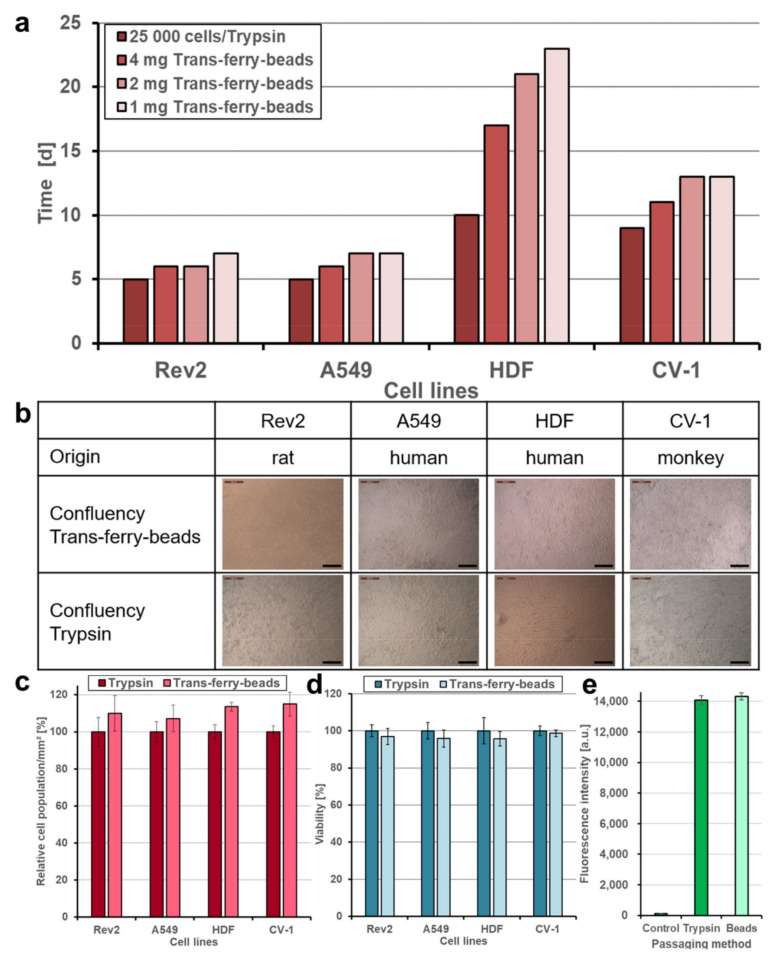
Comparison of trypsinization and trans-ferry-beads as a new passaging method. (**a**) The passaging method using trans-ferry-beads was compared to trypsinization on four cell lines. 25,000 cells were seeded after trypsinization as a reference. (**b**) Confluency of 100% confluency was shown in the optical microscope images for both methods. (**c**) Quantification of cell density (relative population) per mm^2^ of each cell line after reaching 100% confluency depending on the passaging method (*n* = 3 independent experiments). Data are shown as mean ± SD. (**d**) A resazurin assay for determination of the cell metabolic activity after reaching 100% confluency. A factorial ANOVA was used to examine the passaging method’s effect on the cell lines’ metabolic activity (n = 3 independent experiments). No significant difference was detected in the average amount of resorufin metabolized by the cells (F (1) = 0.004, *p* = 0.948), showing that there is no relevant loss in metabolic functions when using the trans-ferry-beads as a passaging method. Data are shown as mean ± SD. (**e**) An independent two-sample *t*-test was used to examine the effect of the passaging method on the gene expression of eYFP in CV-1 cells (*n* = 3 independent experiments). There was no significant effect detected in the average amount of eYFP expressed in the CV-1 cells (t (4) = 0.917, *p* = 0.411), showing no loss in metabolic functions when using the trans-ferry-beads as a passaging method. Data are shown as mean ± SD.

**Figure 7:**
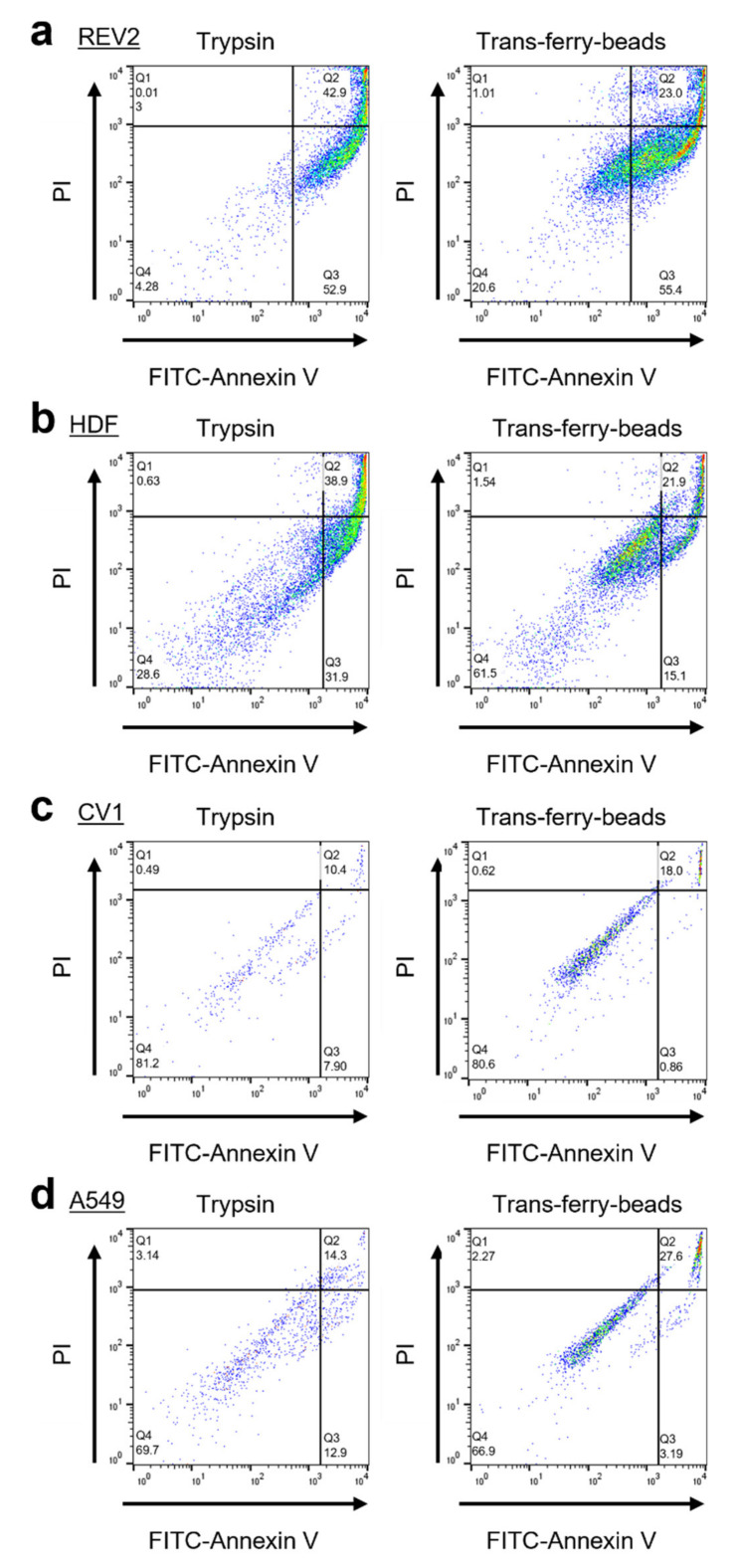
Flow cytometry analyses of passaging methods with trypsin and trans-ferry-beads. Measurements were conducted using a BD FACSCalibur (BD Bioscience, Franklin Lakes, NJ, USA) and evaluated by the software FlowJo (BD Bioscience, Franklin Lakes, NJ, USA). Comparison of living, apoptotic (Annexin V) and death (PI) cells after trysination and passaging using trans-ferry-beads for (**a**) Rev-2, (**b**) HDF, (**c**) CV-1 and (**d**) A549. Q1 indicates the apoptotic cells, Q2 the dead cells and Q3 the viable cells with intact cell membranes.

Subsequently, it was of crucial importance to demonstrate that cells transferred via the trans-ferry-beads are not affected adversely, and unproblematically can repopulate plastic surfaces of fresh cell culture vessels. Thus, this novel passaging/splitting method was compared to the most popular technique based on the use of trypsin for our set of four different cell lines. Normally, after trypsinization, the amount of harvested cells is determined by simple microscopic counting in a Neubauer hemocytometer chamber or measurement in automated cell counters, and afterward defined cell counts are then used to seed the novel population. The optimal cell count for seeding is given by the desired experiment (i.e., the physiological state of growth required for the experiment) and is in addition determined by the growth characteristics of the individual cell line used. One aim of our trans-ferry-bead concept was to obviate such laborious experimental steps such as repeated cell counting, replacing them simply by using different quantities of beads, which then should allow determining the optimal quantity to finally reach a confluent growth in a given time once defined for an individual cell line. With references using 25,000 cells after trypsinization for seeding, it was possible to reach similar time periods for confluency after passaging/splitting with 1–4 mg trans-ferry-beads per well for the Rev2, A549, and CV-1 cell lines, whereas for the HDF cell line significantly extended periods were required (Figure 6a). Since this cell line used in artificial skin models for biomedical testing is known for a relatively slow growth behavior, our results suggest that trans-ferry-bead dependent passaging may be of rather limited benefit for such fastidious cells with respect to time consumption for growth, but undoubtedly allows circumventing additional counting steps also in this case. Moreover, microscopic analysis of the cells after reaching confluency (Figure 6b) demonstrated ordinary and healthy cell morphologies of both the cells split by trypsin treatment and cells transferred by the trans-ferry-beads (Figure 6c). Another important criterion to estimate the quality of a successful cell culture experiment is the resulting cells’ metabolic and physiological integrity, which can be measured as their reductive metabolic activity towards resorufin. In this viability assay, the cells passaged/split via trans-ferry-beads were again indistinguishable from their counterparts originating from trypsinization (factorial ANOVA (F (1) = 0.004, *p* = 0.948) (Figure 6d) finally demonstrating that the amount of 4 mg beads is per se not toxic in this experimental setup using a 6-well plate (i.e., 30-fold larger area), thereby suggesting that the drop in viability observed in the smaller volume of a 96-well plate (see above) really is rather the result of unproductive mechanical influences and spatial limitations than true toxic inhibitory effects. Moreover, the CV-1 cell line harbored a plasmid resulting in heterologous expression of eYFP as a recombinant fluorescent marker. The fluorescence of CV-1 cells and, thus, the amount of recombinant eYFP was independent of the passaging/splitting method used (Figure 6e), demonstrating that cells propagated by trans-ferry-bead transfer were even perfectly fit enough to handle the metabolic burden of recombinant protein production (independent two-sample *t*-test (t (4) = 0.917, *p* = 0.411). To further analyze the cellular apoptosis, senescence and viability a flow cytometric experiment, after cell passaging of A549, CV-1, HDF and Rev-2 cells with trypsin and trans-ferry-beads was performed, respectively. It revealed, that the passaging of cell lines with trans-ferry-beads, compared to the conventional seeding method after trypsination, did not result in a decrease of cell fitness. In contrast, for the cell lines Rev-2 (Figure 7a) and HDF (Figure 7b) even a considerable improvement could be achieved, as after trypsination 43% dead cells were detected compared to 23% after passaging by the trans-ferry-beads based method. Likewise, the number of viable cells in the case of CV-1 (Figure 7c) and A549 (Figure 7d) cell lines similarly ranged from 70% to 80% living cells after passaging with trans-ferry-beads and after trypsination.

This suggests that the trans-ferry-bead mediated procedure for transferring cells is already fully compatible with the requirements of successful cell culture. Furthermore, BSA hydrogels similar to the material presented here for the trans-ferry-beads were shown to offer a broad range of opportunities for further functionalization to improve cell adhesive properties by introducing cells-specific affinity adapter molecules. Efficient binding and subsequent release of cells were achieved by glycosylation of the material in combination with multivalent sugar-binding proteins, and “capture and release” materials for cancer cells were developed based on hydrogels modified with cell-specific aptamers as binding molecules immobilized via hybridization to anchor oligonucleotides crosslinked to the surface of the material [26,27,28,29]. Moreover, dedicated cell adhesive peptides were shown to be a very simple but efficient alternatives to modify BSA hydrogels, thereby improving the molecular attraction of the material for adherent cells via interactions of cellular integrins and the peptide moiety [12]. Modifications of these types can be expected to be readily possible also on the surface of the trans-ferry-beads and, thus, may represent attractive possibilities to further optimize them and to adapt them to applications with more special or fastidious cells as the set of examples used in this study or even to equip the beads with completely new binding specificities for future applications in biomedicine and biotechnology.

## 3. Conclusions

We have described a very simple emulsion-based method for the synthesis of micrometer-scale hydrogel beads via chemical crosslinking of BSA with EDC, which allows a significant simplification of an indispensable and laborious step in the workflows of cell culture laboratories worldwide. The use of our “trans-ferry-beads” can omit traditional harsh trypsinization. When added to different pre-cultivated adherent cell lines, cells efficiently board the particles as passengers and can de-board them after transfer to novel cell culture flasks. This procedure proved to be not harmful to cells, which are perfectly viable and show normal growth behavior afterward. Thus, the BSA hydrogel trans-ferry-beads not only may become more economical as a final cell culture technology product but also may generally replace trypsinization in conventional cell culture, thereby opening new routes for the establishment of optimized and resource-efficient workflows in biological and medical cell culture laboratories.

## 4. Materials and Methods

### 4.1. Material

Bovine serum albumin (BSA), N-(3-dimethylaminopropyl)-N’-ethylcarbodiimide hydrochloride (EDC), 2-(*N*-morpholino)ethanesulfonic acid monohydrate (MES), Triton X-100, and sorbitan monooleate (Span^™^ 80) were purchased from Carl Roth (Carl Roth GmbH und Co. KG, Karlsruhe, Germany). Isopar M^™^ was obtained from Caldic (Caldic Ingredients Deutschland GmbH, Düsseldorf, Germany). Dulbecco’s modified eagle medium (DMEM) supplemented with fetal bovine serum (FBS, 10% (*w*/*v*)), penicillin-streptomycin (100 U mL^−1^, 1% (*w*/*v*)), eagle’s minimum essential medium non-essential amino acids (MEM NEAA) (1% (*w*/*v*)) were received from Life Technologies (Carlsbad, CA, USA). Phosphate-buffered saline (PBS) was purchased from Life Technologies (Carlsbad, CA, USA). Rhodamine B, trypsin-EDTA solution, resazurin, phalloidin Atto 633, and formaldehyde solutions were obtained from Sigma-Aldrich (St. Louis, MO, USA). Rhodamine phalloidin was received from Thermo Fisher Scientific (Waltham, MA, USA). DY-495-NHS-Ester was purchased from Dyomics (Dyomics GmbH, Jena, Germany).

### 4.2. Fabrication of Hydrogel Beads

For the hydrogel mixture, BSA (20% (*w*/*v*)) and EDC (10% (*w*/*v*)) stock solutions were prepared in an MES (0.1 M, pH 5.0) buffer. Isopar M^™^ (16.5 mL) was mixed with Span^™^ 80 (sorbitan monooleate; 50 mg, 117 mmol) to reach a final concentration of 7.1 mmol L^−1^. The mixture was stirred on a magnetic stirrer (Heidolph Instruments, Schwabach, Germany) in a reaction vessel (100 mm, ø 28 mm) with a magnetic stir bar (15 mm, ø 5.0 mm) at 1000 rpm. The hydrogel mixture (1 mL) was prepared by mixing BSA (10% (*w*/*v*)) and EDC (5% (*w*/*v*)) solutions in a 1:1 ratio. For the preparation of fluorescently stained hydrogel beads, the same procedure as above was applied, but DY-495-NHS-Ester solution (5 µL, 1 mg mL^−1^) was added to the BSA (500 µL) solution and mixed with the EDC solution (500 µL). For both stained and non-stained beads, the hydrogel mixture was added immediately to the Isopar M^™^-Span^™^ 80 mixture (continuous phase) before crosslinking took place. The emulsion was stirred for 35 min to ensure complete crosslinking at 1000, 1250, and 1500 rpm, respectively. The beads were separated from the continuous phase by using a folded filter (particle retention 7 µm, ø 185 mm) through gravity flow and were transferred into a reaction vessel containing ethanol (15 mL, 70% (*v*/*v*)) using a spatula. Excessive oil was removed by washing the beads in ethanol. After the beads had been sedimented, the supernatant was discarded. The washing procedure was repeated twice. The beads were stored in ethanol (5 mL, 70% (*v*/*v*)) at 4 °C.

### 4.3. Characterization

An optical microscope (Leica, Wetzlar, Germany) was used to analyze the beads. The average diameter of the beads, expressed as the mean ± standard deviation, was analyzed with the imaging software FIJI. 60 beads per sample were measured. Comparisons between groups were performed with a factorial ANOVA. A value of *p* < 0.05 was considered statistically significant. All measurements were performed in triplicate. The error bars represent the standard deviation. In order to analyze the overall shape of the particles, the beads were stained with rhodamine B solution (20 µL, 1% (*w*/*v*)). The shape of the beads was investigated with an inverted laser scanning microscope (LSM 710, Carl Zeiss, Inc., Oberkochen, Germany) at 514 nm.

### 4.4. Atomic Force Microscopy

Samples for the Atomic Force Microscopy (AFM) measurement were prepared by putting hydrogel beads with DMEM with supplements into a petri dish and letting them adsorb for 15 min. AFM images of the hydrogel beads were obtained with a NanoWizard 4 (JPK BioAFM, Bruker Nano GmbH, Berlin) using Quantitative Imaging measuring mode. The measurements were done at 21 °C in DMEM. To average over a more extensive area in the image for the hydrogel beads, a nanotools biosphere B2000-cont cantilever (nanotools GmbH, Munich) with a nominal spring constant of 0.2 N m^−1^ and a spherical tip with a nominal tip radius of 2 µm was used. As maximum indentation force, 0.3 nN was set. The cantilever approach and retract speed were 35 µm s^−1^ with a sample rate of 100 kHz. The resolution was set to 1.56 µm Pixel^−1^. Young’s modulus was obtained by analyzing the data with the JPK Data Processing Software (JPK BioAFM, Bruker Nano GmbH, Berlin, Germany). The images were bicubic interpolated.

### 4.5. Equilibration of Trans-Ferry-Beads

The beads were aliquoted into a tube, and the ethanol supernatant was removed. For equilibration Dulbecco’s modified eagle medium with supplements (DMEM) was used. The beads were washed with DMEM. After sedimentation of the beads, the supernatant was discarded. The washing procedure was repeated twice for equilibration. The beads were stored in DMEM at 4 °C for the cell culture tests and 37 °C for the storage stability test, respectively. Before use, DMEM was replaced with fresh, prewarmed DMEM.

### 4.6. Cell Culture

The cellular revertant of the SV40 wild-type transformed rat fibroblast cell line SV-52 (Rev2), adenocarcinomic human alveolar basal epithelial cells A549, human dermal fibroblasts HDF and eYFP labelled African green monkey kidney fibroblast CV-1 cells were used for the experiments. Rev2, A549, and HDF were cultured in DMEM supplemented with FBS (10% (*w*/*v*)), MEM NEAA (1% (*w*/*v*)) and penicillin-streptomycin (100 U mL^−1^, 1% (*w*/*v*)) in a 37 °C incubator containing 5% CO_2_. CV-1 were cultured in DMEM supplemented with FBS (10% (*w*/*v*)), penicillin-streptomycin (100 U mL^−1^, 1% (*w*/*v*)), glutamine (2 mM), and geneticin (G418, 4% (*v*/*v*)).

### 4.7. Passaging Adherent Cell Cultures

The cell detachment via trypsinization was performed based on the nature protocol [17]. Briefly, trypsin-EDTA solution, PBS, and cell culture medium with supplements (DMEM) were pre-warmed to 37 °C. The culture media from flasks were removed and discarded. Cells were rinsed with PBS, and the solution was removed. Trypsin-EDTA solution was added and incubated for 5 to 15 min at 37 °C. Once cells were detached, DMEM was added, and the cell suspension was transferred into tubes and centrifuged at 500× *g* for 5 min. After removing the supernatant, the cell pellets were resuspended in DMEM. An appropriate volume was pipetted to the culture flasks or well-plates and incubated at 37 °C containing 5% CO_2_. The cell detachment via trans-ferry-beads was performed by adding an appropriate number of beads to the attached cell lines in a well plate. After incubation at 37 °C containing 5% CO_2_ for 48 h to 72 h (for cell lines Rev2 and A549) and 72 h to 96 h (for cell lines HDF and CV-1), an appropriate volume of the cell loaded beads were transferred via pipette to a new well that contained prewarmed cell culture medium. After another 24 h of incubation at 37 °C containing 5% CO_2_, the cells de-boarded the beads and adhered to the well surface. The beads and cell culture medium were discarded and replaced with fresh prewarmed cell culture medium.

### 4.8. Viability Test Cell Compatibility

A resazurin assay was performed to test the cell viability. Incubation was performed in 2 × 10^4^ cells per well in a 96-well-plate with different bead quantities (0.25, 0.5, 1, 2, and 4 mg) in DMEM with supplements (100 µL) at 37 °C containing 5% CO_2_ for 72 h. After the incubation time, resazurin (20 µL, 0.15 mg mL^−1^) was added to each well and incubated for another 24 h at 37 °C containing 5% CO_2_. The amount of metabolized fluorescent resorufin was measured with a Tecan200 M fluorescence reader (Tecan Group Ltd., Mannedorf, Switzerland) at an excitation wavelength of 535 nm and an emission wavelength of 595 nm. All measurements were performed in triplicate. The error bars represent the standard deviation.

### 4.9. Confocal Laser Scanning Microscopy

In order to visualize the cell growth of Rev2 on the surface of the beads, the µ-slide 8 wells were prepared containing a final volume of 100 µL per well. The cells and beads were washed 4 times with PBS (100 µL). The cells were fixed with formaldehyde (100 µL, 7.4% (*v*/*v*)) for 10 min and washed 4 times with PBS (100 µL). The cells were permeabilized with Triton X-100 (100 µL, 0.2% (*w*/*v*)) for 5 min and washed 4 times with PBS (100 µL). For the first set of confocal images, the cells were stained with a rhodamine-phalloidin solution (5 µL) in PBS (195 µL) for 20 min in the dark. For the second set of confocal images, the cells were stained with cell-specific phalloidin Atto 633 solution (4 µL, 10 nmol) in PBS (196 µL) for 20 min in the dark. After the staining procedure, the cells and beads were washed 4 times with PBS (100 µL). For the second set of confocal images, the fluorescently labeled beads were employed (see above). Adhesion areas were investigated with an inverted laser scanning microscope (LSM 710, Carl Zeiss, Inc., Oberkochen, Germany) at 488, 514, and 633 nm, respectively.

### 4.10. Viability Test after Confluency

A resazurin assay was performed to test the cell viability after reaching confluency. Fresh cell culture medium (1000 µL) was added to the 6-wells after discarding the used medium. Resazurin (200 µL, 0.15 mg mL^−1^) was added to each well and incubated for 4 h at 37 °C containing 5% CO_2_. The amount of metabolized fluorescent resorufin was measured with a Tecan200 M fluorescence reader (Tecan Group Ltd., Männedorf, Switzerland) at an excitation wavelength of 535 nm and an emission wavelength of 595 nm. Comparisons between groups were performed with a factorial ANOVA. A value of *p* < 0.05 was considered statistically significant. All measurements were performed in triplicate. The error bars represent the standard deviation.

### 4.11. Quantification of Cell Density

Cell density was quantified by counting the number of cells in 1 mm^2^ (analyzed area 1000 × 1000 µm) at different selected fields of the photographed wells with the imaging software FIJI. All measurements were performed as a triplicate. The error bars represent the standard deviation.

### 4.12. Cell Lysis and eYFP Extraction

After reaching confluency in a 6-well, the CV-1 cells were washed twice with PBS (1000 µL) and permeabilized with Triton X-100 (1000 µL, 0.2% (*v*/*v*)) for 5 min. 100 µL of the lysate was analyzed with a Tecan200 M fluorescence reader (Tecan Group Ltd., Männedorf, Switzerland) at an excitation wavelength of 535 nm and an emission wavelength of 595 nm. Comparisons between groups were performed with an independent two-sample *t*-test. A value of *p* < 0.05 was considered statistically significant. All measurements were performed in triplicate. The error bars represent the standard deviation.

### 4.13. Flow Cytometric Cytotoxicity Assay

The cell lines were treated with FITC Annexin V Apoptosis Detection Kit I. After passaging the cells via trypsin and trans-ferry-beads, the cells were incubated for 48 h at 37 °C in a 5% CO_2_ to proliferate and reach confluency in 24-well plates. Media was removed and 100 µL serum-free medium was added to stimulate the cells for 24 h. Afterward, cells were resuspended and cooled on ice. 100 µL EDTA/PBS was added for 10 min to harvest cells. 2 mL FACS buffer was added and centrifuged at 1300 rpm for 10 min. The supernatant was discarded, and 1 µL Annexin V-FITC (to dye apoptotic cells) and 5 µL Propidium Iodide (to dye death cells) dye solution in 100 µL 2× binding buffer in dH_2_O were added. Cells were resuspended and incubated for 15 min at RT in the dark. 200 µL of 1× binding buffer in dH_2_O were added. Cells were analyzed with a BD FACSCalibur (BD Bioscience, Becton, 1 Becton Drive, NJ, USA).

## Data Availability

Not applicable.
